# Clinical validation of AI‐assisted contouring in prostate radiation therapy treatment planning: Highlighting automation bias and the need for standardized quality assurance

**DOI:** 10.1002/acm2.70425

**Published:** 2025-12-19

**Authors:** Najmeh Arjmandi, Ahmed Reza Sebzari, Fatemeh Molaei, Saeid Rezaei, Maryam Rezaie‐Yazdi, Malihe Rezaie‐Yazdi

**Affiliations:** ^1^ Department of Radiology School of Paramedical Sciences Gerash University of Medical Sciences Gerash Iran; ^2^ Department of Internal Medicine School of Medicine Birjand University of Medical Sciences Birjand Iran; ^3^ Department of Radiation Oncology Iran Mehr Hospital Birjand University of Medical Sciences Birjand Iran; ^4^ Radiation Oncology Research Center Cancer Research Institute Tehran University of Medical Sciences Tehran Iran; ^5^ Physics Unit, Department of Radiation Oncology Iran Mehr Hospital Birjand University of Medical Sciences Birjand Iran

**Keywords:** Auto‐contouring, Automation bias, Deep learning, Inter‐observer variability, Intra‐observer variability, Quality assurance, Radiotherapy

## Abstract

**Purpose:**

This study evaluated the impact of a commercial AI‐assisted contouring tool on intra‐ and inter‐observer variability in prostate radiation therapy and assessed the dosimetric consequences of geometric contour differences.

**Methods:**

Two experienced radiation oncologists independently delineated clinical target volume (CTV) and organs at risk (OARs) for prostate cancer patients. Manual contours (C_man_) and AI‐generated contours (C_AI_) were compared with adjusted AI contours (C_AI,adj_). A consensus reference (C_ref_) served as the benchmark. To evaluate clinical impact, treatment plans were recalculated and replanned on each contour set under identical beam geometries to assess dose–volume histogram (DVH) parameters.

**Results:**

AI‐assisted contouring significantly improved both intra‐ and inter‐observer agreement. Inter‐observer analysis revealed that the Dice similarity coefficient (DSCs) for CTV increased from 0.78 (± 0.11) for C_man_ to 0.89 (± 0.09) for C_AI, adj_. Similarly, intra‐observer analysis revealed that both oncologists showed significantly higher DSCs for C_AI, adj_ compared to C_man_. A thorough geometric comparison to the C_ref_ revealed that while adjustments to C_AI_ improved accuracy, they generally did not surpass C_man_ for CTV and rectum. Dosimetric analyses demonstrated that, under fixed plan geometry, both C_man_ and C_AI,adj_ contours yielded lower planning target volume (PTV) D95% values compared with C_ref_, whereas after replanning, all plans met institutional criteria with no clinically significant differences among contour sets.

**Conclusion:**

AI‐assisted contouring in prostate radiotherapy reduced intra‐ and inter‐observer variability and improved contouring consistency. However, C_AI, adj_ did not consistently surpass C_man_, especially for the CTV and rectum, where automation bias or selective clinical acceptance may have influenced edits. Fixed‐plan recalculations revealed dose differences from minor geometric deviations. These findings underscore the importance of structured quality assurance (QA) and human oversight to mitigate automation bias while leveraging AI's efficiency. The single‐institution design with two oncologists and one AI software limits generalizability, underscoring the need for multi‐observer validation.

## 1 INTRODUCTION

Accurate volume delineation on computed tomography (CT) images is a critical yet challenging component of radiotherapy (RT) planning. This process is not only time‐intensive but also prone to errors, which can substantially affect treatment efficacy and patient safety.^1^, ^2^ Persistent inter‐ and intra‐observer variability in contouring remains a major concern, even among experienced radiation oncologists, leading to significant inconsistencies in the definition of target volumes and organs at risk (OARs) across multiple cancer types.^3^, ^4^, ^5^ Notably, the extent of contouring variability often surpasses uncertainties related to organ motion and patient setup, thereby undermining treatment precision.^6^, ^7^, ^8^, ^9^, ^10^

Traditional strategies to mitigate this variability—such as site‐specific atlases, consensus guidelines, and rigorous quality assurance (QA) protocols—have shown limited effectiveness in standardizing contouring practices. Consequently, there is a pressing need for advanced solutions.^11^, ^12^

Deep learning algorithms have emerged as promising tools for auto‐segmentation of clinical target volume (CTV) and OARs across diverse tumor sites including head and neck, prostate, and breast cancers. Indeed, recent surveys indicate that nearly half of clinicians have incorporated DL‐based auto‐contouring into clinical workflows, reflecting growing confidence in these technologies.^9^, ^13^

However, the lack of standardized validation and QA frameworks for AI tools in radiotherapy impedes reliable performance assessment and comparison of different models. This inconsistency poses risks for equitable clinical outcomes and challenges the development of robust, trustworthy AI systems. Although automated contouring can enhance efficiency, rigorous human oversight remains indispensable.^10^ Medical physicists are responsible for comprehensive quality assurance, and radiation oncologists maintain ultimate clinical responsibility for treatment structures.^14^ However, despite progress in these established protocols and the attention given to auto‐segmentation in thoracic CT and head and neck MRI by the AAPM's Grand Challenges in 2017^15^ and 2019^16^, an evaluation of commercial prostate auto‐contouring systems is currently lacking. This study aims to partially address this gap by evaluating the impact of AI‐assisted contouring on intra‐ and inter‐physician consistency in prostate radiotherapy.

## 2 MATERIALS AND METHODS

### 2.1 Patient data

This study analyzed noncontrast CT scans from 17 randomly selected patients with localized, early‐stage prostate cancer who had received RT within the past 2 years and had not undergone prior prostatectomy, rectal spacer placement, or femoral implant placement. The planning CT images were acquired using a GE LightSpeed 16 scanner, covering the region from the sacrum to below the ischiatic tuberosities. Patients were scanned in a supine position with full bladders, and the acquisition parameters were 120 kVp, 180–300 mAs, pitch 1, and soft kernel reconstruction. The resulting 512 * 512 matrix images had in‐plane/through‐plane resolutions of 0.82–0.97 mm and 3–5 mm, respectively, with CT numbers ranging from −985 to 3890 HU. Initial window width and level settings varied across patients (WW: 250–1130 HU; WL: −100 to 310 HU).

### 2.2 Contouring workflows

In this study, three contouring methods were employed for each patient across two distinct phases, separated by a 2‐month interval.

Phase 1: Manual Contouring (C_man_)

During the first phase, two experienced radiation oncologists, each with over 10 years of clinical experience, independently performed manual contouring from scratch. They delineated the CTV, which included the entire prostate and its capsule, as well as the OARs, specifically the rectum, bladder, right femoral head (RFH), and left femoral head (LFH). This manual contouring was conducted using a 3D PCRT treatment planning system (TPS) (V6.0.2.15, TRF Group, Zaragoza, Spain).

Each oncologist performed contouring for each patient twice, with a minimum interval of 2 weeks between sessions to minimize recall bias. To ensure the independence of their work, the oncologists were blinded to their own prior contours as well as those of their colleagues.

To further reduce potential bias, the oncologists were informed that they would be contouring images for a total of 30 patients; however, they worked exclusively with the same 15 patients in both sessions. To ensure anonymity and facilitate accurate comparisons, we anonymized and sequentially numbered the images from the first contouring session. These images were then duplicated for the second session, utilizing different numbers for the duplicates while maintaining the original sequence.

The patients' images were presented to the oncologists in a carefully structured manner, ensuring a minimum interval of two weeks between each session for each patient. This design provided the oncologists with a consistent set of images, reinforcing the necessity of the 2‐week interval between contouring sessions. This controlled approach aimed to mitigate the influence of memory on contouring decisions.

It is noteworthy that two patients were excluded from the study due to identifiable features related to their conditions (extensive rectal dilation and the presence of Foley urethral catheter). Prior to the contouring process, both patients and physicians were anonymized to maintain confidentiality. The oncologists utilized a brush tool with slice interpolation—a standard practice in clinical settings—to delineate the contours. They also had optional access to standard TPS tools, including window/level adjustments, zoom functionalities, and sagittal/coronal views, to facilitate accurate contouring.

Phase 2: AI‐Assisted Contouring

The second phase incorporated fully automated AI‐based contouring (C_AI_) and AI‐based contouring that was subsequently reviewed and adjusted by a radiation oncologist (C_AI, adj_) 2 months after the first phase. This 2‐month gap between the first and second phases allowed oncologists to approach each contouring task with a fresh perspective, minimizing the risk of bias associated with prior experiences with the same patient.

The C_AI_ contours of the prostate and OARs were generated for 15 patients using Limbus Contouring Software (V1.7.0‐B3, Limbus AI Inc., Regina, SK, Canada) 2 months after manual contouring. The software employs U‐Net‐based convolutional neural networks, each trained to segment a specific anatomy, with post‐processing steps including z‐plane cutoffs, smoothing outlier removal, and slice interpolation. A prostate RT model, trained using contours of the bladder, rectum, femoral heads, and prostate, was used.

The C_AI_ contours were imported as DICOM files into the 3D PCRT TPS for review and validation. For the C_AI, adj_, oncologists independently reviewed and modified the C_AI_ contours as needed while being blinded to the initial manual contours. The design for modifying auto‐contours was similar to that of the first phase. Each oncologist modified the auto‐contours for each patient twice, ensuring a minimum interval of two weeks between sessions to minimize recall bias.

Similar to the manual contouring done from scratch, the oncologists were informed that they would be reviewing modified auto‐contours for a total of 30 patients; however, they worked exclusively with the same 15 auto‐contours generated for each patient in both sessions. The patients' images and corresponding C_AI_ contours were presented to the oncologists in a carefully structured manner, maintaining the 2‐week interval between each session for each patient. All modifications utilized brush and eraser tools integrated within the TPS.

### 2.3 Reference standard consensus delineation

Following all individual contouring activities, an unbiased reference standard was established. This was achieved by convening a panel of three clinical experts, including one radiologist and two prostate radiation oncologists, who collaboratively performed consensus segmentations of the prostate and OARs on the axial CT image series. This consensus delineation (C_ref_) provided a validated benchmark for subsequent quantitative assessments and comparison of contours across manual and AI‐assisted methods.

### 2.4 Contour evaluation

DICOM RT structure sets were converted to binary NIfTI label maps for quantitative analysis. Geometric metrics were calculated using Python package seg‐metrics (V1.2.8).

Contour volume comparisons were performed using the Dice similarity coefficient (DSC) and Hausdorff distances (HD). The DSC quantifies the spatial overlap between two contours, with higher values indicating better agreement. The HD, representing the maximum nearest‐neighbor Euclidean distance between volumes, measures boundary discrepancies. To mitigate the influence of outliers, we employed the 95th percentile HD (HD95); lower values signify improved contour agreement.^17^

This study evaluated the impact of automated contouring on individual radiation oncologist decision‐making by comparing DSC and HD for each oncologist between: (1) C_AI_ and C_man_; and (2) C_AI_ and C_AI, adj_.

The impact of automated contouring on intra‐ and inter‐observer agreement were also assessed using these metrics. For intra‐observer agreement, each oncologist performed two series independent manual delineations (C_man, S1_, C_man, S2_) and two independent adjustments of automated contours (C_AI, adj, S1_, C_AI, adj, S2_). The DSC for intra‐observer evaluation of manual contours is calculated based on following formula:

(1) $$DSC=2*\left(C_{\mathrm{man},\;S1}\cap C_{\mathrm{man},\;S2})/(C_{\mathrm{man},\;S1}+C_{\mathrm{man},\;S2}\right)$$

where C_man, S1_ represents the manual contours from the first series and C_man, S2_ represents the manual contours from the second series.

Inter‐observer agreement was also evaluated using the same metrics, comparing both C_man_ and C_AI, adj_ between two oncologists to determine the impact of automated contouring on inter‐observer variability.

Statistical analysis used two‐tailed Wilcoxon–Mann–Whitney tests for nonparametric data and independent samples *t*‐tests for parametric data, with significance set at *p* < 0.05. All analyses were conducted using SPSS (V26.0.0).

### 2.5 Dosimetric impact analysis

This addition was performed to strengthen the clinical interpretation of geometric findings. A dosimetric analysis was conducted to evaluate the clinical implications of contour variability. For each radiation oncologist, nine patients were randomly selected. All selected cases were anonymized prior to plan generation and dose recalculation to maintain blinding during analysis. For each patient, identical clinician‐determined anisotropic margins were applied to all three CTV contour sets (C_man_, C_AI,adj_, and C_ref_) to generate the corresponding planning target volumes (PTVs). Margin size varied between patients according to clinical judgment, typically ranging from 5–10 mm, with a posterior margin of 5–6 mm to account for rectal proximity.

All treatment plans were generated using the treatment planning system for an Elekta Compact linear accelerator (Elekta, Stockholm, Sweden) equipped with an 80‐leaf multileaf collimator (MLC) and delivering a 6 MV photon beam. Dose calculations were performed using the superposition algorithm with a 3‐mm calculation grid. A prescription dose of 70 Gy in 35 fractions was applied to the PTV. Because the intensity‐modulated radiation therapy (IMRT) option was unavailable on the institutional TPS, all treatment plans were generated using three‐dimensional conformal radiotherapy (3D‐CRT).

Conventional four‐field box radiotherapy—comprising opposed anterior–posterior and lateral beams—served as the baseline plan geometry. “Hot spots” within the PTV were constrained not to exceed 105% of the prescription dose. Two complementary planning strategies were implemented:

Dose recalculation without re‐optimization: The original clinical plan, optimized on C_ref_, was recalculated on the alternative contour sets (C_man_ and C_AI,adj_) without modification to isolate the direct dosimetric effect of geometric variability.

Replanning with identical beam geometry and optimization objectives: To reflect clinical practice, for each contour set, new plans were generated using identical beam arrangements and optimization goals to evaluate achievable dosimetry after planner compensation.

Dose–volume histogram (DVH) parameters were extracted and compared across plan sets. Evaluated metrics included PTV D_95%_, D_2%_, D_max_, and D_mean_, as well as OAR parameters for the rectum and bladder (D_max_, D_mean_, V_50_, and V_65_) and femoral heads (D_mean_). Table 1 summarizes the applied clinical dose criteria.

**TABLE 1:** Clinical dose objectives and evaluation criteria used for 3D‐CRT prostate treatment planning.

	**3D‐CRT**
Volume	Dose criteria
**CTV**	100% of the CTV receives at least 100% of the prescribed dose (PD).
**PTV**	> 95% of the PTV receives at least 95% of the PD.
**Bladder**	V50 < 50% V65 < 50%
**Rectum**	V50 < 50% V65 < 25%
**Femoral heads**	Mean Dose < 37 Gy

Abbreviations: 3D CRT, 3D‐conformal radiotherapy; CTV, clinical target volume; PTV, planning target volume.

## 3 RESULTS

### 3.1 Geometric evaluation

#### 3.1.1 Intra‐physician evaluation

**FIGURE 1:**
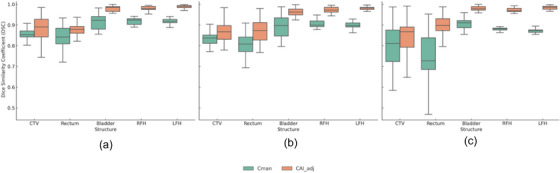
Box plots illustrating (a) the impact of the AI‐based contouring system on intra‐observer agreement for the first radiation oncologist; (b) the impact on intra‐observer agreement for the second radiation oncologist; and (c) the impact on inter‐observer agreement between the two radiation oncologists.

The impact of the AI‐based contouring system on intra‐observer agreement is presented in Table 2 and illustrated in Figure 1 using box plots. For both radiation oncologists, the DSC values for the CTV, rectum, bladder, RFH, and LFH were consistently higher in the C_AI, adj_ group, indicating better spatial overlap and agreement.

**TABLE 2:** The impact of AI‐based contouring system on intra‐observer agreement.

		Intra‐ physician Evaluation (15 patients)									
		**First Radiation oncologist**									
		**CTV**		**Rectum**		**Bladder**		**RFH**		**LFH**	
		**DSC**	**HD95**	**DSC**	**HD95**	**DSC**	**HD95**	**DSC**	**HD95**	**DSC**	**HD95**
**C_man, S1, S2_**		0.86 (± 0.03)	6.3 (± 1.8)	0.84 (± 0.06)	8.0 (± 7.7)	0.92 (± 0.04)	4.4 (± 2.1)	0.92 (± 0.02)	4.7 (± 2.0)	0.92 (± 0.01)	4.1 (± 1.1)
**C_AI, adj_, _S1, S2_**		0.90 (± 0.08)	2.6 (± 1.1)	0.88 (± 0.05)	2.4 (± 3.1)	0.98 (± 0.02)	1.7 (± 0.9)	0.99 (± 0.02)	0.3 (± 0.5)	0.99 (± 0.01)	0.4 (± 0.2)
**p‐value**		0.044*	0.039*	0.037*	0.019*	0.018*	0.011*	0.020*	≤0.001*	0.022*	≤0.001*
		**Second Radiation oncologist**									
		**CTV>**		**Rectum**		**Bladder**		**RFH**		**LFH**	
		**DSC**	**HD95**	**DSC**	**HD95**	**DSC**	**HD95**	**DSC**	**HD95**	**DSC**	**HD95**
**C_man, S1, S2_**		0.83 (± 0.12)	5.7 (± 3.2)	0.88 (± 0.05)	6.3 (± 5.3)	0.94 (± 0.03)	3.3 (± 0.5)	0.92 (± 0.01)	2.4 (± 0.6)	0.91 (± 0.03)	3.4 (± 0.9)
**C_AI, adj, S1, S2_**		0.92 (± 0.11)	3.2 (± 2.9)	0.91 (± 0.10)	3.4 (± 2.1)	0.98 (± 0.04)	1.2 (± 0.5)	0.97 (± 0.04)	1.5 (± 0.5)	0.97 (± 0.02)	0.9 (± 0.4)
***p*‐value**		≤ 0.001*	0.041*	0.053	0.034*	0.033*	0.028*	0.019*	0.058	0.023*	0.009*

Abbreviations: CAI, adj, S1, S2, adjusted automated contours across two sessions; Cman, S1, S2, manual contours across two sessions; CTV, clinical target volume; DSC, dice similarity coefficient; HD95, 95th percentile Hausdorff distances; LFH, left femoral head; RFH, right femoral head.

**FIGURE 2:**
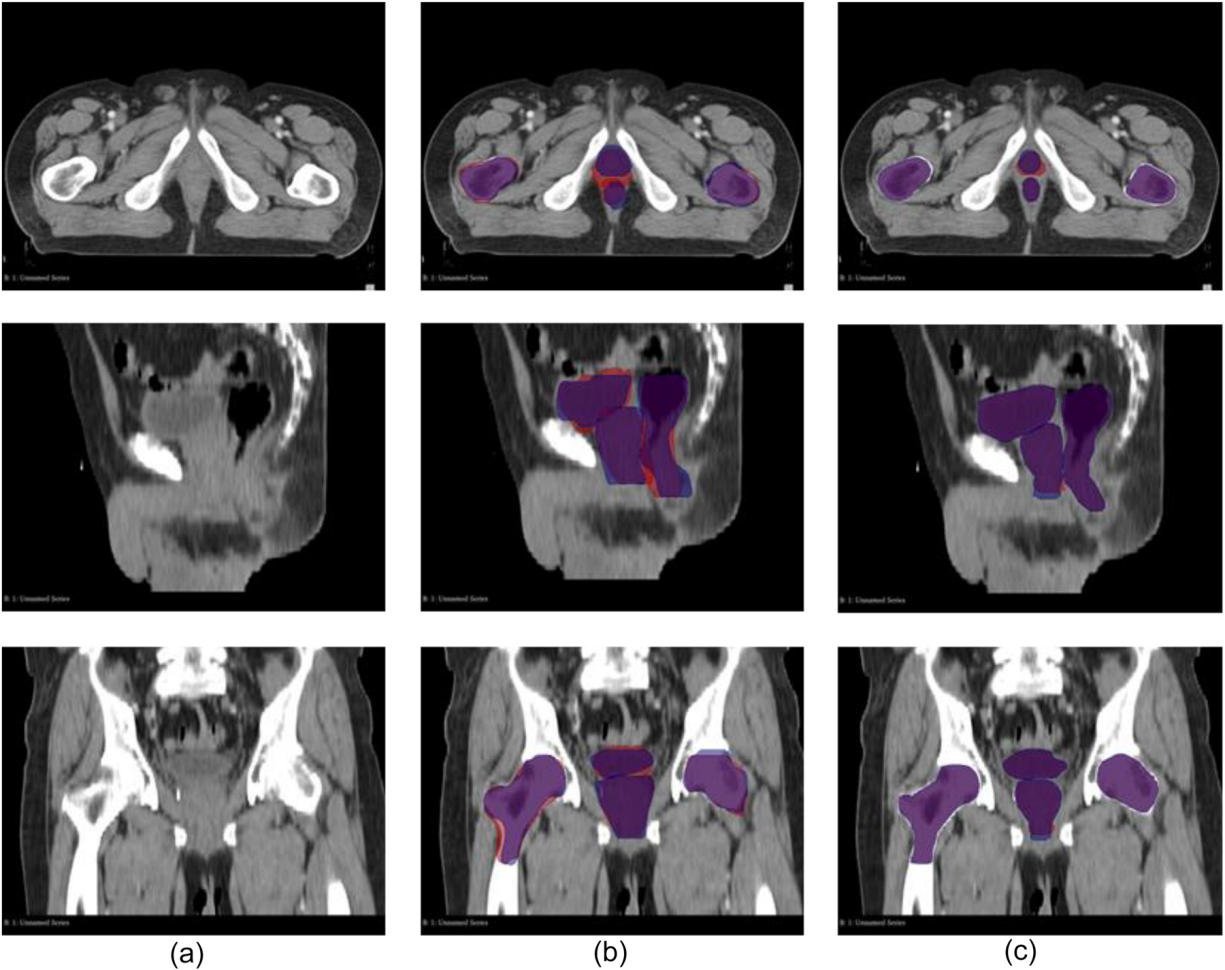
Impact of AI‐assisted contouring on intra‐observer agreement. Panel (a) Representative patient CT images (transverse, sagittal, and coronal views). Panel (b) Overlay of two independent manual contouring sessions (C_man, S1 overlay on_ C_man, S2_, S1 in blue; S2 in red), illustrating intra‐observer variability. Panel (c) Overlay of two independent adjusted AI‐assisted contouring sessions (C_AI, adj, S1 overlay on_ C_AI, adj, S2_, S1 in blue; S2 in red), demonstrating improved spatial overlap for the RT structures, indicative of enhanced intra‐observer agreement. The significantly greater spatial overlap in Panel (c) highlights the improved consistency achieved with AI‐assisted contouring.

For the first radiation oncologist, the DSC for CTV improved from 0.86 (± 0.03) in C_man_ to 0.90 (± 0.08) in the C_AI, adj_ (*p* = 0.044). Similarly, the HD95 decreased from 6.3 (± 1.8) mm to 2.6 (± 1.1) mm, demonstrating a significant reduction in boundary discrepancies. The second radiation oncologist exhibited comparable results, with a DSC increase from 0.83 (± 0.12) to 0.92 (± 0.11) for CTV (*p* < 0.001) and a reduction in HD95 from 5.7 (± 3.2) to 3.2 (± 2.9) mm. This trend was consistent across all structures evaluated, including the rectum, bladder, and femoral heads. These quantitative findings are supported by the visual inspection of Figure 2, which demonstrates substantially greater spatial overlap of contours in the AI‐assisted workflow.

#### 3.1.2 Inter‐physician evaluation

**FIGURE 3:**
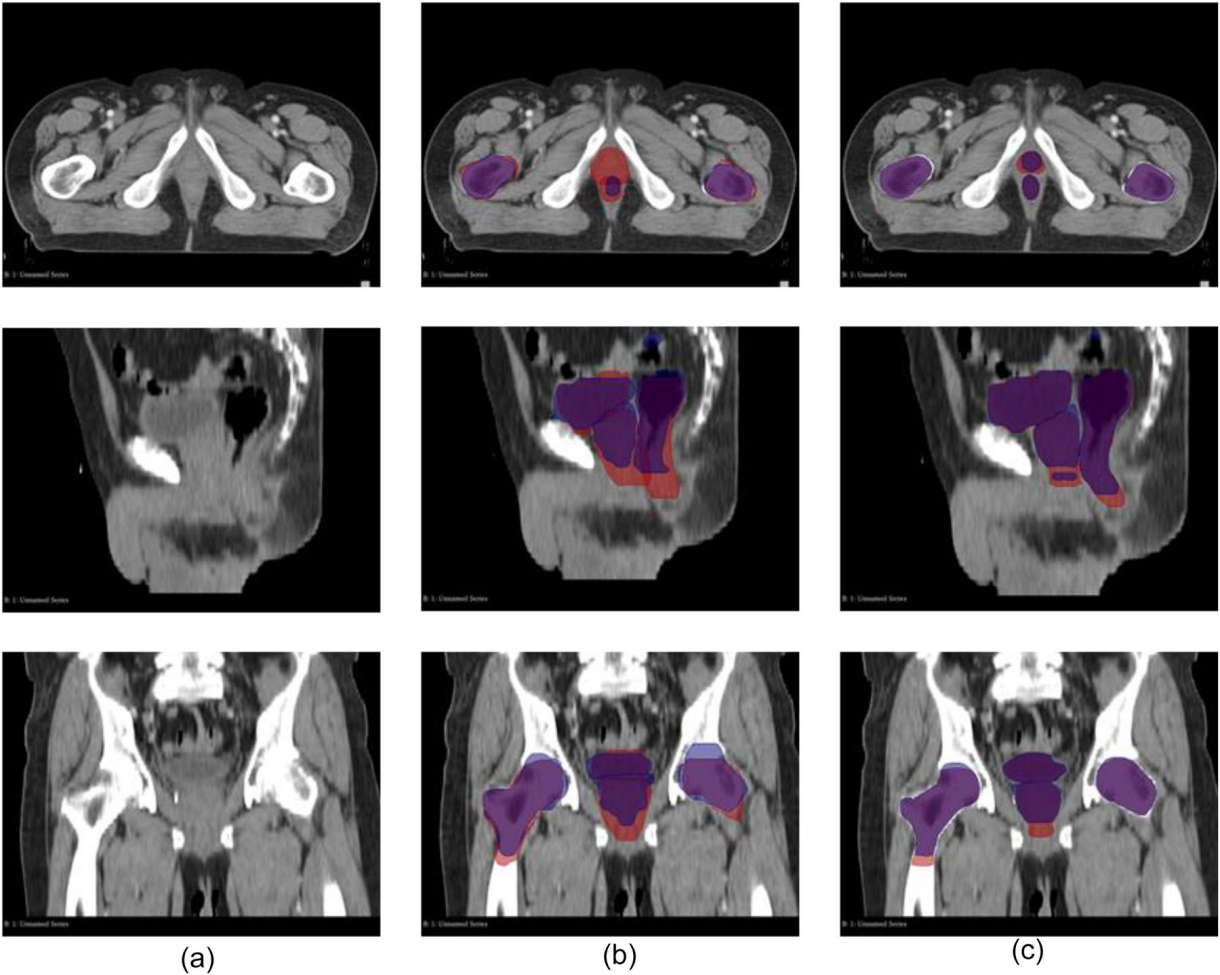
Impact of AI‐assisted contouring on inter‐observer agreement. Panel (a) Representative patient CT images (transverse, sagittal, and coronal views). Panel (b) Overlay of two independent manual contours (oncologist 1: blue; oncologist 2: red), illustrating inter‐observer variability. Panel (c) Overlay of two independent AI‐assisted adjusted contours (oncologist 1: blue; oncologist 2: red), demonstrating improved spatial overlap for all RT structures. The increased overlap in Panel (c) highlights the improved consistency achieved with AI‐assisted contouring.

As summarized in Table 3 and illustrated in Figure 1, the AI‐based contouring system substantially improved inter‐observer agreement. The DSC for the CTV increased from 0.78 (± 0.11) for C_man_ to 0.89 (± 0.09) for C_AI, adj_, with a statistically significant *p*‐value of < 0.001. This trend was consistent across all evaluated RT structures. The HD95 also demonstrated significant reductions, indicating improved boundary agreement. For example, the HD95 for CTV decreased from 15.9 (± 2.1) mm in the C_man_ group to 4.7 (± 2.2) mm in the C_AI_,_adj_ group (*p* < 0.001). Similar reductions were observed for the rectum (from 20.1 to 3.6 mm), bladder (from 3.9 to 1.7 mm), RFH (from 5.7 to 1.2 mm), and LFH (from 5.2 to 0.7 mm), all with *p*‐values < 0.05. This enhanced agreement is further visually supported by Figure 3, showing significantly improved contour overlap with the introduction of AI‐assisted contours.

**TABLE 3:** The impact of AI‐based contouring system on inter‐observer agreement. The reported results are average of DSCs and the HD95s for all possible pairings within each group.

		Inter‐ physician evaluation (15 patients)									
		**First Radiation oncologist vs Second Radiation oncologist**									
		**CTV**		**Rectum**		**Bladder**		**RFH**		**LFH**	
		**DSC**	**HD95**	**DSC**	**HD95**	**DSC**	**HD95**	**DSC**	**HD95**	**DSC**	**HD95**
**C_man, O1, O2_**		0.78 (± 0.11)	15.9 (± 2.1)	0.76 (± 0.11)	20.1 (± 2.7)	0.91 (± 0.03)	3.9 (± 4.2)	0.88 (± 0.01)	5.7 (± 3.2)	0.87 (± 0.01)	5.2 (± 4.1)
**C_AI, adj, O1, O2_**		0.89 (± 0.09)	4.7 (± 2.2)	0.90 (± 0.05)	3.6 (± 1.2)	0.99 (± 0.02)	1.7 (± .9)	0.97 (± 0.01)	1.2 (± 1.0)	0.98 (± 0.01)	0.7 (± 1.2)
**p‐value**		≤ 0.001*	≤ 0.001*	≤ 0.001*	≤ 0.001*	≤0.001*	0.042*	≤ 0.001*	0.013*	≤0.001*	≤0.001*

Abbreviations: CAI, adj, O1, O2, adjusted automated contours across two oncologists; Cman, O1, O2, manual contours across two oncologists; CTV, clinical target volume; DSC, dice similarity coefficient; HD95, 95th percentile Hausdorff distances; LFH, left femoral head; RFH, right femoral head.

### 3.2 The impact of automated contouring on individual radiation oncologist decision‐making

The dataset comprised 15 patients for the assessment of intra‐ and inter‐observer variability. To enhance the statistical power and clinical robustness of the analysis, an additional 10 patients were included exclusively for this sub‐analysis.

**FIGURE 4:**
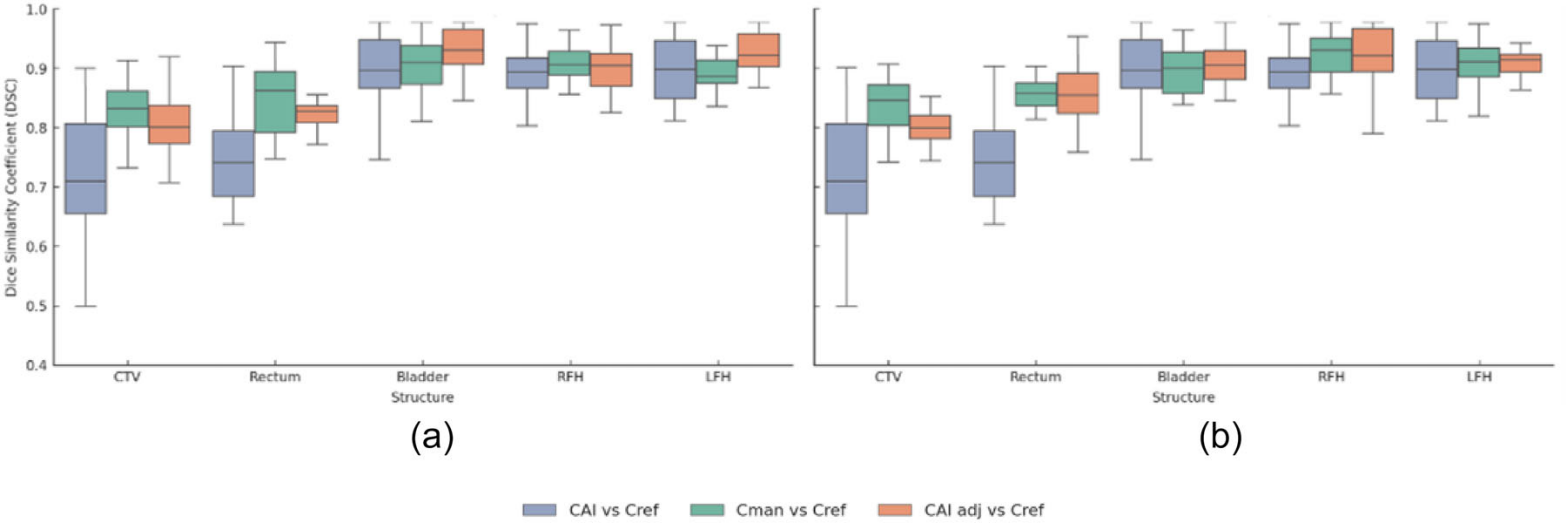
Box plots showing the impact of automated contouring on decision‐making for (a) the first radiation oncologist and (b) the second radiation oncologist.

Table 4 provides a detailed comparison of C_AI_, C_man_, and C_AI, adj_ against a C_ref_ for both radiation oncologists across critical prostate RT structures, as illustrated in the Figure 4 box plots.

**TABLE 4:** The impact of automated contouring on individual radiation oncologist decision‐making. The reported results are average of DSCs and the HD95s for all possible pairings within each group.

		Evaluation of automated contouring on oncologist decisions (25 patients)									
		**CTV**		**Rectum**		**Bladder**		**RFH**		**LFH**	
		**DSC**	**HD95**	**DSC**	**HD95**	**DSC**	**HD95**	**DSC**	**HD95**	**DSC**	**HD95**
**C_AI_ vs C_ref_**		0.74 (± 0.13)	9.7 (± 3.3)	0.75 (± 0.07)	8.3 (± 5.0)	0.93 (± 0.05)	4.1 (± 5.2)	0.91 (± 0.04)	3.8 (± 4.1)	0.93 (± 0.06)	4.2 (± 1.6)
		**First Radiation oncologist**									
**C _man_ vs C_ref_**		0.85 (± 0.06)	5.81 (± 5.1)	0.84 (± 0.07)	4.1 (± 5.9)	0.93 (± 0.05)	2.1 (± 2.6)	0.93 (± 0.03)	3.6 (± 2.1)	0.93 (± 0.04)	4.9 (± 1.3)
**C _AI, adj_ vs C_ref_**		0.81 (± 0.07)	8.3 (± 0.1)	0.81 (± 0.04)	6.8 (± 3.0)	0.94 (± 0.06)	3.4 (± 1.5)	0.93 (± 0.04)	0.2 (± 0.1)	0.95 (± 0.06)	0.3 (± 0.1)
**p‐value**		0.030*	0.044*	0.036*	0.057	0.113	0.175	0.561	0.024*	0.154	0.005*
		**Second Radiation oncologist**									
**C _man_ vs C_ref_**		0.83 (± 0.05)	5.4 (± 2.7)	0.86 (± 0.03)	2.8 (± 3.4)	0.91 (± 0.06)	4.5 (± 1.9)	0.94 (± 0.03)	1.3 (± 0.9)	0.94 (± 0.04)	3.8 (± 2.1)
**C _AI, adj_ vs C_ref_**		0.80 (± 0.07)	11.8 (± 2.0)	0.86 (± 0.05)	5.6 (± 2.3)	0.94 (± 0.05)	1.1 (± 1.3)	0.95 (± 0.06)	2.4 (± 1.1)	0.93 (± 0.03)	1.8 (± 0.9)
***p*‐value**		0.045*	0.008*	0.612	0.044*	0.034*	0.048*	0.083	0.210	0.341	0.087

Abbreviations: CAI, adj, adjusted automated contours; CAI, automated AI‐based contours; Cman, manual contours; Cref, reference standard consensus contours; CTV, clinical target volume; DSC, dice similarity coefficient; HD95, 95th percentile Hausdorff distances; LFH, left femoral head; RFH, right femoral head.

Initial AI contours for the CTV achieved a moderate DSC of 0.74 (± 0.13) with a corresponding HD95 of 9.7 mm (± 3.3). In contrast, C_man_ from the first radiation oncologist demonstrated superior accuracy with a DSC of 0.85 (± 0.06) and HD95 of 5.8 mm (± 5.1). Adjusted AI contours improved upon the initial AI output, reaching a DSC of 0.81 (± 0.07) and HD95 of 8.3 mm (± 0.1), though accuracy remained generally lower than fully manual delineations. A similar pattern was seen for the rectum, where C_AI_ contours had a DSC of 0.75 (± 0.07) and HD95 of 8.3 mm (± 5.0); C_man_ exhibited higher agreement (DSC 0.84 ± 0.07; HD95 4.1 ± 5.9), and adjusted AI contours were intermediate (DSC 0.81 ± 0.04; HD95 6.8 ± 3.0). For the bladder, AI contours showed high overlap (DSC 0.93 ± 0.05; HD95 4.1 ± 5.2), manual contours were comparable (DSC 0.93 ± 0.05; HD95 2.1 ± 2.6), and adjustments slightly improved agreement (DSC 0.94 ± 0.06; HD95 3.4 ± 1.5). The right and left femoral heads followed a similar trend: AI contours had good DSC values (0.91 and 0.93) but larger HD95 values (3.8–4.2 mm), manual contours showed better boundary conformity (HD95 3.6–4.9 mm), and adjusted contours reached the highest concordance (DSC 0.93–0.95; HD95 as low as 0.2–0.3 mm). For the second radiation oncologist, comparable results were observed.

### 3.3 Dosimetric evaluation

To assess the clinical relevance of geometric contour differences, dosimetric analyses were performed under two complementary planning scenarios: (1) recalculation on fixed plans without re‐optimization and (2) replanning using identical beam geometries and optimization objectives. The corresponding quantitative results are summarized in Tables 5 and 6, respectively. To further assess differences between groups, statistical analyses were performed using the Wilcoxon signed‐rank test, with *p‐*values < 0.05 considered statistically significant.

**TABLE 5:** Quantitative dosimetric evaluation of 3D‐CRT plans for nine patients based on fixed‐plan recalculation.

3D‐CRT plans of nine patients (recalculation of fix plan)							
Volume	Oncologist	Dosimetric index	C_ref_ Mean ± SD	C_man_ Mean ± SD	C_AI, adj_ Mean ± SD	*p*‐value C_ref_‐C_man_	*p*‐value C_ref_‐C_AI,adj_
**PTV**	O1 O2	D_95%_ /cGy D_2%_ /cGy D_max_ /cGy D_mean_ /cGy D_95%_ /cGy D_2%_ /cGy D_max_ /cGy D_mean_ /cGy	6995 ± 28 7440 ± 37 7535 ± 31 7210 ± 32 6998 ± 35 7491 ± 46 7576 ± 51 7227 ± 34	4625 ± 3791 7422 ± 12 7535 ± 32 6111 ± 1751 5722 ± 2196 7490 ± 43 7576 ± 50 6882 ± 439	5895 ± 1851 7421 ± 53 7520 ± 50 7061 ± 186 6740 ± 293 7492 ± 47 7576 ± 50 7150 ± 108	0.0085* 0.8086 0.9923 0.0249* 0.0190* 0.9889 0.9998 0.0454*	0.0205* 0.8022 0.8148 0.2127 0.0159* 0.9902 0.9998 0.3071
**Bladder**	O1 O2	V_5000 cGy_ /% V_6500 cGy_ /% D_max_ /cGy D_mean_ /cGy V_5000 cGy_ /% V_6500 cGy_ /% D_max_ /cGy D_mean_ /cGy	15.73 ± 13.37 11.43 ± 9.40 7304 ± 127 2980 ± 124 21.40 ± 13.43 13.75 ± 7.15 7318 ± 77 2848 ± 1464	14.74 ± 12.01 10.60 ± 9.11 7274 ± 99 2849 ± 17 22.47 ± 14.03 15.92 ± 9.43 7352 ± 28 2838 ± 1417	15.99 ± 12.45 11.75 ± 10.75 7288 ± 113 3024 ± 86 20.71 ± 9.80 13.73 ± 5.07 6847 ± 836 2797 ± 1231	0.2115 0.3621 0.3143 0.2029 0.1922 0.0924 0.4436 0.5021	0.2459 0.7120 0.7249 0.3001 0.2490 0.9171 0.0421* 0.2158
**Rectum**	O1 O2	V_5000 cGy_ /% V_6500 cGy_ /% D_max_ /cGy D_mean_ /cGy V_5000 cGy_ /% V_6500 cGy_ /% D_max_ /cGy D_mean_ /cGy	19.86 ± 13.12 12.85 ± 8.99 7442 ± 141 3439 ± 1446 15.31 ± 2.96 8.19 ± 1.94 7343 ± 198 2493 ± 310	22.31 ± 12.30 15.15 ± 7.79 7449 ± 126 3374 ± 1779 12.93 ± 6.07 7.35 ± 5.76 7310 ± 254 2514 ± 160	26.60 ± 14.58 20.70 ± 10.74 7447 ± 125 3991 ± 1793 19.37 ± 3.57 11.73 ± 3.00 7329 ± 195 3444 ± 1093	0.0912 0.1111 0.8904 0.1021 0.0740 0.1241 0.7744 0.2546	0.0350* 0.0241* 0.9152 0.0430* 0.0489* 0.0671 0.7942 0.0226*
**RFH**	O1 O2	D_mean_ /cGy D_mean_ /cGy	3379 ± 958 3032 ± 327	3087 ± 439 3001 ± 377	3296 ± 559 3117 ± 266	0.0561 0.4142	0.1945 0.1683
**LFH**	O1 O2	D_mean_ /cGy D_mean_ /cGy	3382 ± 950 2995 ± 447	3097 ± 463 2939 ± 484	3340 ± 599 3079 ± 489	0.3411 0.3817	0.3022 0.4817

Abbreviations: 3D‐CRT, three‐dimensional conformal radiotherapy; CAI, adj, AI‐based adjusted contour; Cman, manul contour; Cref, reference contour; LFH, left femoral head; PTV, planning target volume; RFH, right femoral head.

**TABLE 6:** Quantitative dosimetric evaluation of 3D‐CRT plans for nine patients based on re‐optimized plans.

3D CRT Plans of Nine Patients (re‐optimized plans)							
Volume	Oncologist	Dosimetric index	C_ref_ Mean ± SD	C_man_ Mean ± SD	C_AI,adj_ Mean ± SD	*p*‐value C_ref_‐C_man_	*p*‐value C_ref_‐C_AI,adj_
**PTV**	O1 O2	D_95%_ /cGy D_2%_ /cGy D_max_ /cGy D_mean_ /cGy D_95%_ /cGy D_2%_ /cGy D_max_ /cGy D_mean_ /cGy	6984 ± 32 7470 ± 56 7567 ± 50 7212 ± 32 7011 ± 23 7460 ± 47 7544 ± 44 7225 ± 36	6909 ± 79 7434 ± 78 7526 ± 47 7170 ± 74 6996 ± 21 7498 ± 64 6586 ± 951 7247 ± 51	7056 ± 105 7484 ± 26 7601 ± 28 7261 ± 62 7023 ± 80 7446 ± 48 7525 ± 53 7225 ± 66	0.6184 0.7869 0.7323 0.7142 0.8909 0.9101 0.0327* 0.4541	0.7250 0.8212 0.3148 0.6970 0.9252 0.9392 0.8988 0.9974
**Bladder**	O1 O2	V_5000 cGy_ /% V_6500 cGy_ /% D_max_ /cGy D_mean_ /cGy V_5000 cGy_ /% V_6500 cGy_ /% D_max_ /cGy D_mean_ /cGy	21.94 ± 17.33 14.36 ± 11.00 7316 ± 114 3405 ± 860 15.19 ± 4.97 10.82 ± 3.63 7305 ± 95 2423 ± 843	17.02 ± 6.47 11.15 ± 4.28 7279 ± 234 2739 ± 173 15.12 ± 3.99 10.98 ± 3.19 7338 ± 156 2451 ± 578	15.62 ± 10.92 10.05 ± 6.60 7359 ± 127 3125 ± 260 13.06 ± 4.07 9.04 ± 2.78 7303 ± 84 1702 ± 332	0.0727 0.3925 0.4443 0.0102* 0.9714 0.9021 0.9206 0.5021	0.0352* 0.0512 0.4992 0.0548 0.4100 0.8774 0.9725 0.0323*
**Rectum**	O1 O2	V_5000 cGy_ /% V_6500 cGy_ /% D_max_ /cGy D_mean_ /cGy V_5000 cGy_ /% V_6500 cGy_ /% D_max_ /cGy D_mean_ /cGy	14.42 ± 4.00 8.11 ± 1.01 7327 ± 206 3234 ± 1154 20.76 ± 12.24 12.93 ± 9.10 7457 ± 80 3250 ± 1419	16.86 ± 10.76 10.91 ± 9.88 7352 ± 171 3257 ± 700 22.51 ± 7.47 13.81 ± 4.62 7531 ± 41 3421 ± 1168	13.39 ± 7.14 8.71 ± 5.19 7398 ± 187 2758 ± 1253 13.23 ± 5.19 7.13 ± 2.40 7389 ± 62 2991 ± 1105	0.2352 0.2817 0.8914 0.7025 0.5141 0.2491 0.2045 0.1289	0.4536 0.9041 0.7959 0.0404* 0.0329* 0.0361* 0.1928 0.0469*
**RFH**	O1 O2	D_mean_ /cGy D_mean_ /cGy	3275 ± 1036 3136 ± 168	3206 ± 532 4449 ± 1724	3187 ± 740 3160 ± 393	0.6919 0.0315*	0.3105 0.7828
**LFH**	O1 O2	D_mean_ /cGy D_mean_ /cGy	3271 ± 1028 3694 ± 1640	3203 ± 712 3111 ± 471	3147 ± 800 3583 ± 748	0.6021 0.0440*	0.4702 0.2099

Abbreviations: 3D‐CRT, three‐dimensional conformal radiotherapy; CAI, adj, AI‐based adjusted contour; Cman, manul contour; Cref, reference contour; LFH, left femoral head; PTV, planning target volume; RFH, right femoral head.

**FIGURE 5:**
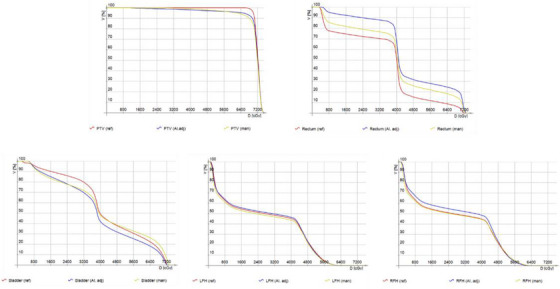
DVHs for a representative patient planned with 3D‐CRT, illustrating dose distributions to the PTV and OARs, including the rectum, bladder, and femoral heads.

In the fixed‐plan recalculation scenario (Table 5), dose distributions were recomputed on C_man_ and C_AI,adj_ contours using the same beam parameters optimized for C_ref_. Across both oncologists, PTV coverage (D_95%)_ was significantly reduced, indicating clinically meaningful underdosage in the recalculated C_man_ and C_AI,adj_ plans compared with C_ref_, and this reduction was also statistically significant (*p* = 0.008–0.021). However, no significant differences were observed for high‐dose indices (D_2%_ and D_max_) across contour sets (*p* > 0.80). Rectum and bladder dose–volume parameters, although clinically acceptable, showed some statistical differences between contour sets. For the bladder, mean and high‐dose parameters showed no statistically significant differences between contour sets, except for a single comparison that reached significance (O2 D_max_, *p* = 0.042). Rectum dose–volume parameters, particularly V_50_ and V_65_, showed a tendency toward higher doses in C_AI, adj_ compared with C_ref_. These differences achieved statistical significance in several cases (e.g., O1 V_50_, *p* = 0.035; O1 V_65_, *p* = 0.024; O2 V_50_, *p* = 0.049; O2 D_mean_, *p* = 0.023). Figure 5 presents the DVH for a representative case.

In the replanning scenario (Table 6), where treatment plans were re‐optimized on each contour set, all dosimetric parameters met institutional clinical criteria. PTV coverage metrics showed no statistically significant differences among C_ref_, C_man_, and C_AI,adj_ (*p* > 0.6). For the OARs, most bladder and rectum dose–volume parameters were not significantly different from C_ref_ following replanning. However, a few indices showed minor but statistically significant differences—specifically bladder D_mean_ for first oncologist (*p* = 0.010) and rectum D_mean_ for second oncologist (*p* = 0.047). Despite these findings, all variations remained within acceptable clinical limits. For the femoral heads, both right and left, mean doses remained below 35 Gy across all contour sets, well within institutional constraints.

## 4 DISCUSSION

Automated contouring tools offer a more efficient alternative to expert manual delineation but still require rigorous review and validation. Although these systems can improve workflow efficiency, the ultimate responsibility for contour accuracy and patient safety remains with human experts—medical physicists ensuring QA and radiation oncologists maintaining clinical accountability.^14^ While the AAPM's Grand Challenges in 2017^15^ and 2019^16^ addressed auto‐segmentation in thoracic CT and head and neck MRI, respectively, a comprehensive evaluation of commercially available systems for prostate remains lacking. This study partly fills this gap by evaluating the impact of AI‐assisted contouring on intra‐ and inter‐physician consistency in prostate RT, acknowledging the potential time savings while highlighting the critical need for bias mitigation strategies.

Intra‐observer analysis (Table 2) revealed significantly improved contouring consistency when using C_AI, Adj_ compared to C_man_, as evidenced by substantially higher DSCs and lower HD. This improvement highlights the potential of AI integration in clinical workflows to reduce intra‐observer variability. Furthermore, inter‐observer analysis (Table 3) demonstrated a similar enhancement in contouring agreement when utilizing AI‐generated contours. The significant increases in DSC and decreases in HD95 indicate that AI‐assisted contouring facilitates greater consensus among radiation oncologists in volume delineation. Across all structures (CTV, rectum, bladder, and bilateral femoral heads), the superior performance of C_AI, adj_ compared to C_man_ was consistently observed in both intra‐ and inter‐observer comparisons (*p* < 0.05 in most cases; *p* < 0.001 in many instances), reinforcing the value of AI‐assisted contouring in improving the reproducibility and reliability of contouring practices. These findings align with previous research showing AI‐assisted contouring reduces inter‐ and intra‐observer variability in prostate (Palazzo et al.),^8^ breast (Choi et al.),^18^ in endometrial cancer (Ma et al.),^19^ and other cancers (Hoque et al.).^20^ While automated segmentation demonstrably reduces intra‐ and inter‐observer variability,^8^, ^18^ the degree of improvement varies considerably depending on factors such as observer expertise, model selection, and anatomical site.^21^

A thorough comparison against the reference contours (Table 4) reveals notable constraints of the initial AI‐generated contours. For the CTV and rectum, the raw AI contours showed only moderate accuracy, with DSC ranging from 0.73 to 0.75 and HD95 as high as 8.2 mm. In contrast, manual contours consistently demonstrated higher precision, with DSC values between 0.82 and 0.85 and notably lower HD95, sometimes approaching 4.9 mm. Encouragingly, when clinicians reviewed and edited AI contours, accuracy for structures such as the bladder and femoral heads surpassed that of manual delineations, underscoring AI's capacity to improve contour reproducibility for anatomically well‐defined organs. However, this benefit did not extend to the CTV and rectum, where adjusted AI contours remained less accurate than completely manual contours. This pattern may indicate the influence of automation bias, whereby clinicians, possibly prioritizing workflow efficiency or perceiving AI‐generated contours as sufficiently reliable, tended to accept and modify AI outputs—even when those were objectively inferior to their manual work.

Although the observed tendency to accept or minimally modify AI‐generated contours has often been interpreted as a manifestation of automation bias, an alternative explanation warrants consideration. Prior research suggests that decision‐makers interacting with algorithmic outputs may not simply unconsciously defer to them but may instead consciously adopt them when judged to be “good enough,” balancing perceived adequacy with efficiency or time constraints.^22^, ^23^ Clinicians may therefore be exercising deliberate clinical judgment—consciously determining that AI‐assisted contours are sufficiently accurate for clinical purposes and that minor geometric deviations are unlikely to produce meaningful dosimetric differences. In this light, such behavior reflects an intentional balance between precision and efficiency, consistent with real‐world clinical workflows and time pressures. This interpretation highlights the nuanced nature of human–AI interaction, suggesting that what is often characterized as automation bias may, in certain contexts, represent a rational and informed clinical decision. Future studies incorporating qualitative methods, such as decision‐tracking or post‐contouring interviews, could help differentiate unconscious bias from conscious, efficiency‐driven judgment.

These observations underscore a critical challenge in the implementation of AI‐assisted contouring: the need for robust QA protocols and training frameworks to mitigate automation bias. While the convenience and efficiency gains offered by AI are undeniable, the potential for clinicians to inadvertently accept inaccurate AI suggestions necessitates a careful balance between automation and human oversight. The lack of a clear definition of “clinical acceptance” in the context of AI‐assisted contouring further complicates this issue. This concern is underscored by Lee et al.’s findings, which, while showing improved accuracy metrics (DSC and HD95) following the implementation of auto contouring, emphasize the crucial role of robust QA and risk management strategies in mitigating automation bias and optimizing clinical workflow.^10^

To ensure the safe and effective implementation of AI‐assisted contouring, a multifaceted QA strategy is essential. This strategy should encompass both case‐specific assessments, which involve human review to address clinical risks, and routine evaluations that monitor overall model performance using updated benchmark datasets.^10^, ^24^, ^25^ The integration of automated QA tools and user‐friendly interfaces that leverage statistical and machine learning methods can further enhance the identification of outliers and the quantification of segmentation accuracy.^25^, ^26^ As highlighted by Nealon et al., tracking the frequency of manual corrections can serve as an effective indicator of performance degradation, whether stemming from the AI model or user error.^27^

The current landscape of auto‐contouring evaluation remains fragmented, with no standardized metrics for assessing clinical utility.^17^ McQuinlan et al.’s findings underscored the importance of incorporating geometric overlap metrics into physician review workflows to address clinician perception bias, which significantly influences the acceptance of automated contours.^28^ The diversity of metrics utilized in previous studies, as illustrated by Robert et al., further emphasizes the need for consensus on specific evaluation criteria to facilitate meaningful comparisons across different systems.^29^ Moreover, the 2019 ESTRO guidelines advocate for a combined qualitative and quantitative evaluation of auto‐contouring systems, aiming for accuracy that aligns with typical inter‐ or intra‐observer variability.^30^ However, the absence of specific metric recommendations within these guidelines hinders the establishment of a standardized framework for evaluating AI‐assisted contouring tools. Mitigating automation bias requires a concerted effort to enhance user transparency regarding AI reasoning processes,^31^ reinforce human accountability,^32^ and communicate uncertainties associated with AI outputs.^33^ Additionally, targeted training on the reliability of AI systems within specific clinical contexts is crucial to empower clinicians to make informed decisions when integrating AI into their practice.^34^, ^35^

Our dosimetric analysis provided complementary insight into the clinical implications of geometric differences. Recalculation of the original, C_ref_‐optimized plan on alternative contours isolated the direct dosimetric effect of geometric differences (Table 5), whereas replanning on each contour set evaluated the real‐world clinical implications under routine planning conditions (Table 6). Overall, we observed the expected pattern: In fixed‐plan recalculations, both C_man_ and C_AI,adj_ plans exhibited reduced PTV coverage (D_95%_) relative to C_ref_, confirming that geometric discrepancies translate to measurable dose differences when plans are not re‐optimized. However, after replanning on each contour set, these differences largely disappeared and produced only small residual differences for most DVH indices. These outcomes align with multiple prior reports that geometric contour differences do not always translate into clinically meaningful dosimetric degradations once the planner can compensate.^36^, ^37^

From the geometric analysis, we initially expected that manual contour–based plans would yield dose distributions more consistent with the reference than AI‐adjusted plans under fixed‐geometry conditions. However, the findings from Table 5 did not entirely confirm this expectation. The fixed‐plan results did not uniformly penalize the AI‐adjusted contours; in several cases, the C_AI, adj_ contours produced slightly higher PTV dose indices (e.g., D_95%_ and D_mean_) than the manual contours, despite exhibiting poorer geometric agreement with the reference. Detailed inspection of dose distributions revealed the geometric mechanism behind this apparent paradox. The AI‐adjusted segmentations—derived from the auto‐contouring tool and subjected to only minimal oncologist modification—systematically underestimated prostate volume in the mid‐portion of the CTV, whereas manual contours tended to over‐expand in this region. Consequently, when the plan optimized for the reference contour was recalculated on these differing volumes, the smaller AI‐based prostate contours encompassed a greater fraction of the prescribed dose in the mid‐portion of the target, yielding comparable or slightly higher PTV coverage despite inferior geometric similarity. For instance, in a representative case (Figure 5) where the CTV DSC was 0.842 for the manual versus reference contours and 0.793 for the AI, adj versus reference contours, the recalculated PTV D95 values were 6197 and 6509cGy, respectively, compared with 6996 cGy in the reference plan. This finding illustrates how spatial variations in contour shape—not merely overall geometric overlap—can meaningfully influence dosimetric outcomes.

Another noteworthy observation from the fixed‐plan recalculation analysis was the elevated rectal dose associated with the C_AI,adj_ contours compared with the reference contours, although absolute differences remained within clinically acceptable limits. As presented in Table 5, several rectal dose–volume parameters, particularly V50, V65, and D_mean_, were significantly higher for C_AI,adj_ (*p* < 0.05). Further evaluation of isodose distributions clarified the cause: the AI‐adjusted contours, derived from minimally edited auto‐segmentations, tended to include a slightly larger proximal rectal volume than the reference contours. This subtle geometric expansion leading to higher recorded rectal doses.

For the other OARs—the bladder, LFH, and RFH—dosimetric parameters showed no statistically significant differences between the AI‐adjusted and reference contours, indicating that dose metrics for these organs are largely insensitive to contour variability under fixed beam geometry. This outcome aligns with the geometric analysis, in which AI‐assisted contours demonstrated close spatial agreement with the reference for the bladder and femoral heads.

Collectively, these findings emphasize that the dosimetric impact of AI‐generated contours is both organ‐specific and geometry‐dependent. While AI‐assisted contouring achieved dosimetric equivalence for structures with well‐defined anatomical boundaries (e.g., bladder and femoral heads), the prostate and rectum demonstrated greater sensitivity to contour variability. This suggests that automation bias—whether conscious or unconscious—can influence both geometric and dosimetric outcomes for these target structures, whereas its impact appears negligible for organs at risk with clearer boundaries. These results underscore the need for case‐by‐case quality assurance and clinician oversight, particularly in regions where small geometric deviations may produce clinically meaningful dosimetric effects.

Our dosimetric findings from re‐optimized plans align with previous reports showing that geometric differences between manual and AI‐assisted CTV contours rarely lead to clinically significant dosimetric variations once re‐optimization is performed.^36^, ^37^, ^38^, ^39^ In contrast, our fixed‐plan recalculation demonstrated that when no replanning is applied, even minor geometric discrepancies—whether from manual or AI‐adjusted contours relative to the reference—can produce measurable dosimetric changes. This observation is consistent with Dinç et al., who likewise used fixed‐plan evaluation of the DirectORGANS algorithm and reported that, despite strong geometric agreement, subtle prostate boundary deviations resulted in significant dosimetric differences between auto‐ and manual contours.^40^

Although IMRT is typically regarded as the standard of care in prostate radiotherapy, our previous evaluation^41^ using an AI‐assisted contouring platform demonstrated consistent dosimetric trends for both 3D‐CRT and IMRT. Despite IMRT's higher conformity and steeper gradients, no significant differences in the relative dosimetric impact of contouring method were observed, even at higher prescription doses (78 Gy vs. 70 Gy), supporting the validity of the present 3D‐CRT–based analysis.

An important consideration relates to the generalizability of these findings. This study was conducted at a single institution, involving two oncologists and one commercial AI contouring software. While this controlled design allowed a rigorous evaluation of intra‐ and inter‐observer variability, it limits the ability to fully capture the heterogeneity of clinical practice. The extent of automation bias and the perceived adequacy of AI‐generated contours are likely influenced by multiple factors, including the baseline performance of the AI model, user interface design, and individual clinician experience. For instance, high‐quality initial contours or efficient editing tools may encourage greater reliance on AI assistance, whereas suboptimal outputs may prompt more manual correction. These dynamics underscore that automation bias is not a fixed attribute but a context‐dependent process shaped by both algorithmic and human factors. Consequently, although our findings align with prior literature, they should be interpreted with caution, and future multicenter and multivendor studies are needed to evaluate how differences in AI model performance, software interface design, and user experience influence contour accuracy, clinician behavior, and workflow efficiency.

Our nine‐patient dosimetric subset provided representative data for analysis; however, larger and more diverse sample sizes are necessary to validate these findings. Future research should also extend to other disease sites to establish the broader clinical applicability of AI‐assisted contouring. Finally, contouring times were not recorded in this study, preventing a direct evaluation of the accuracy–efficiency trade‐off. In our prior work^41^ with the same AI‐assisted tool, contour generation was approximately 78% faster than manual delineation. While this efficiency gain may contribute to automation bias—encouraging clinicians to accept or minimally adjust AI‐generated contours even when inferior—this relationship remains hypothetical and should be tested prospectively in studies that capture both efficiency and accuracy metrics.

## 5 CONCLUSION

AI‐assisted contouring demonstrates strong potential to enhance the consistency and efficiency of prostate radiation therapy treatment planning. In this study, the integration of a commercial AI contouring tool significantly reduced intra‐ and inter‐observer variability. However, the findings also highlight the nuanced nature of clinician–AI interaction. For anatomically complex structures such as the CTV and rectum, adjusted AI contours did not consistently outperform manual delineations, suggesting residual limitations related to both model accuracy and user interaction. The observed tendency of clinicians to accept or minimally modify AI‐generated contours may arise from both automation bias and deliberate clinical judgment—where experienced users consciously balance acceptable geometric deviations against workflow efficiency. Recognizing this distinction is critical for developing balanced frameworks for AI integration, combining quality assurance with user trust and workflow optimization.

Dosimetrically, fixed‐plan analyses revealed that geometric deviations can produce measurable dose variations, particularly for target structures and rectum. However, after plan re‐optimization, all contour sets achieved clinically equivalent dose distributions, aligning with prior studies indicating that planner intervention can largely neutralize geometric discrepancies. These results suggest that AI‐assisted contouring is clinically viable once supported by robust review and QA protocols.

In clinical deployment, AI contouring should therefore be accompanied by structured, case‐level QA procedures, systematic monitoring of correction frequency, and institution‐specific validation to ensure performance consistency. Broader multi‐observer, multi‐institutional studies across different AI platforms are essential to establish generalizability and to clarify how automation bias and clinician experience interact to shape real‐world performance. Ultimately, safe and effective AI integration in radiotherapy will depend on balancing automation‐driven efficiency with informed human oversight.

## AUTHOR CONTRIBUTIONS

**Najmeh Arjmandi**: Study conception; image processing and analysis; manuscript writing. **Ahmed Reza Sebzari and Fatemeh Molaei**: Organ contouring and manuscript writing. **Saeid Rezaei**: Data collection and manuscript writing. **Maryam & Malihe Rezaie‐Yazdi**: Supervision. All authors contributed to results interpretation and manuscript revision.

## CONFLICT OF INTEREST STATEMENT

The authors declare no conflicts of interest.

## ETHICS STATEMENT

This study was reviewed and approved by the Ethics Committee of Birjand University of Medical Sciences.

## Data Availability

The datasets used and analyzed in this study are available from the corresponding author upon reasonable request.

## References

[1] Polymeri E , Johnsson ÅA , Enqvist O , et al. Artificial intelligence‐based organ delineation for radiation treatment planning of prostate cancer on computed tomography. Adv Radiat Oncol. 2024;9(3):101383. doi:10.1016/j.adro.2023.101383 38495038 10.1016/j.adro.2023.101383 PMC10943520

[2] Matoska T , Patel M , Liu H , Beriwal S . Review of deep learning based autosegmentation for clinical target volume: current status and future directions. Adv Radiat Oncol. 2024;9(5):101470. doi:10.1016/j.adro.2024.101470 38550365 10.1016/j.adro.2024.101470 PMC10966174

[3] Rong Y , Chen Q , Fu Y , et al. NRG oncology assessment of artificial intelligence deep learning–based auto‐segmentation for radiation therapy: current developments, clinical considerations, and future directions. Int J Radiat Oncol. 2024;119(1):261‐280. doi:10.1016/J.IJROBP.2023.10.033 10.1016/j.ijrobp.2023.10.033 PMC11023777 37972715

[4] Zhong AY , Lui AJ , Kuznetsova S , et al. Clinical impact of contouring variability for prostate cancer tumor boost. Int J Radiat Oncol Biol Phys. 2024;120(4):1024‐1031. doi:10.1016/j.ijrobp.2024.06.007 38925224 10.1016/j.ijrobp.2024.06.007

[5] Hope A , Mundis M , Sonke JJ , et al. Three discipline collaborative radiation therapy (3DCRT) special debate: aI structure segmentation is better than clinician contouring for both OARs and targets. J Appl Clin Med Phys. 2025;26(7):e70183. doi:10.1002/ACM2.70183 40660865 10.1002/acm2.70183 PMC12260264

[6] Segedin B , Petric P . Uncertainties in target volume delineation in radiotherapy—are they relevant and what can we do about them?. Radiol Oncol. 2016;50(3):254‐262. doi:10.1515/RAON‐2016‐0023 27679540 10.1515/raon-2016-0023 PMC5024655

[7] Jameson MG , Holloway LC , Vial PJ , Vinod SK , Metcalfe PE . A review of methods of analysis in contouring studies for radiation oncology. J Med Imaging Radiat Oncol. 2010;54(5):401‐410. doi:10.1111/J.1754‐9485.2010.02192.X 20958937 10.1111/j.1754-9485.2010.02192.x

[8] Palazzo G , Mangili P , Deantoni C , et al. Real‐world validation of artificial intelligence‐based computed tomography auto‐contouring for prostate cancer radiotherapy planning. Phys imaging Radiat Oncol. 2023;28:100501. doi:10.1016/J.PHRO.2023.100501 37920450 10.1016/j.phro.2023.100501 PMC10618761

[9] Hindocha S , Zucker K , Jena R , et al. Artificial intelligence for radiotherapy auto‐contouring: current use, perceptions of and barriers to implementation. Clin Oncol. 2023;35(4):219‐226. doi:10.1016/J.CLON.2023.01.014 10.1016/j.clon.2023.01.014 36725406

[10] Lee BM , Kim JS , Chang Y , et al. Experience of implementing deep learning‐based automatic contouring in breast radiation therapy planning: insights from over 2000 cases. Int J Radiat Oncol Biol Phys. 2024;119(5):1579‐1589. doi:10.1016/j.ijrobp.2024.02.041 38431232 10.1016/j.ijrobp.2024.02.041

[11] Huq MS , Fraass BA , Dunscombe PB , et al. The report of Task Group 100 of the AAPM: application of risk analysis methods to radiation therapy quality management. Med Phys. 2016;43(7):4209‐4262. doi:10.1118/1.4947547 27370140 10.1118/1.4947547 PMC4985013

[12] Vandewinckele L , Claessens M , Dinkla A , et al. Overview of artificial intelligence‐based applications in radiotherapy: recommendations for implementation and quality assurance. Radiother Oncol. 2020;153:55‐66. doi:10.1016/J.RADONC.2020.09.008 32920005 10.1016/j.radonc.2020.09.008

[13] Stas D , De Kerf G , Claessens M , et al. Incorporating indirect MRI information in a CT‐based deep learning model for prostate auto‐segmentation. Radiother Oncol. 2025;206:110806. doi:10.1016/j.radonc.2025.110806 39988305 10.1016/j.radonc.2025.110806

[14] Zanca F , Brusasco C , Pesapane F , Kwade Z , Beckers R , Avanzo M . Regulatory aspects of the use of artificial intelligence medical software. Semin Radiat Oncol. 2022;32(4):432‐441. doi:10.1016/J.SEMRADONC.2022.06.012 36202445 10.1016/j.semradonc.2022.06.012

[15] Yang J , Veeraraghavan H , Armato SG , et al. Autosegmentation for thoracic radiation treatment planning: a grand challenge at AAPM 2017. Med Phys. 2018;45(10):4568‐4581. doi:10.1002/MP.13141 30144101 10.1002/mp.13141 PMC6714977

[16] Cardenas CE , Mohamed ASR , Yang J , et al. Head and neck cancer patient images for determining auto‐segmentation accuracy in T2‐weighted magnetic resonance imaging through expert manual segmentations. Med Phys. 2020;47(5):2317‐2322. doi:10.1002/MP.13942 32418343 10.1002/mp.13942 PMC7322982

[17] Mackay K , Bernstein D , Glocker B , Kamnitsas K , Taylor A . A review of the metrics used to assess auto‐contouring systems in radiotherapy. Clin Oncol (R Coll Radiol). 2023;35(6):354‐369. doi:10.1016/J.CLON.2023.01.016 36803407 10.1016/j.clon.2023.01.016

[18] Choi MS , Chang JS , Kim K , et al. Assessment of deep learning‐based auto‐contouring on interobserver consistency in target volume and organs‐at‐risk delineation for breast cancer: implications for RTQA program in a multi‐institutional study. The Breast. 2024;73:103599. doi:10.1016/J.BREAST.2023.103599 37992527 10.1016/j.breast.2023.103599 PMC10700624

[19] Ma CY , Zhou JY , Xu XT , et al. Deep learning‐based auto‐segmentation of clinical target volumes for radiotherapy treatment of cervical cancer. J Appl Clin Med Phys. 2022;23(2):e13470. doi:10.1002/ACM2.13470 34807501 10.1002/acm2.13470 PMC8833283

[20] Hoque SMH , Pirrone G , Matrone F , et al. Clinical Use of a commercial artificial intelligence‐based software for autocontouring in radiation therapy: geometric performance and dosimetric impact. Cancers (Basel). 2023;15(24):5735. doi:10.3390/cancers15245735 38136281 10.3390/cancers15245735 PMC10741804

[21] Baroudi H , Brock KK , Cao W , et al. Automated contouring and planning in radiation therapy: what is “clinically acceptable”?. Diagnostics (Basel, Switzerland). 2023;13(4):667. doi:10.3390/DIAGNOSTICS13040667 36832155 10.3390/diagnostics13040667 PMC9955359

[22] Alon‐Barkat S , Busuioc M . Human–AI interactions in public sector decision making: “automation bias” and “selective adherence” to algorithmic advice. J Public Adm Res Theory. 2023;33(1):153‐169. doi:10.1093/JOPART/MUAC007

[23] Horowitz MC , Kahn L . Bending the automation bias curve: a study of human and ai‐based decision making in national security contexts. Int Stud Q. 2024;68(2):sqae020. doi:10.1093/ISQ/SQAE020

[24] Claessens M , Oria CS , Brouwer CL , et al. Quality assurance for AI‐Based applications in radiation therapy. Semin Radiat Oncol. 2022;32(4):421‐431. doi:10.1016/J.SEMRADONC.2022.06.011 36202444 10.1016/j.semradonc.2022.06.011

[25] De Biase A , Sijtsema NM , Janssen T , Hurkmans C , Brouwer C , van Ooijen P . Clinical adoption of deep learning target auto‐segmentation for radiation therapy: challenges, clinical risks, and mitigation strategies. BJR|Artificial Intell. 2024;1(1):ubae015. doi:10.1093/BJRAI/UBAE015 10.1093/bjrai/ubae015 PMC13045716 42064392

[26] Fan M , Wang T , Lei Y , et al. Evaluation and failure analysis of four commercial deep learning‐based autosegmentation software for abdominal organs at risk. J Appl Clin Med Phys. 2025;26(4):e70010. doi:10.1002/ACM2.70010 39946266 10.1002/acm2.70010 PMC11969109

[27] Nealon KA , Han EY , Kry SF , et al. Monitoring variations in the use of automated contouring software. Pract Radiat Oncol. 2024;14(1):e75‐e85. doi:10.1016/j.prro.2023.09.004 37797883 10.1016/j.prro.2023.09.004

[28] McQuinlan Y , Brouwer CL , Lin Z , et al. An investigation into the risk of population bias in deep learning autocontouring. Radiother Oncol. 2023;186:109747. doi:10.1016/J.RADONC.2023.109747 37330053 10.1016/j.radonc.2023.109747

[29] Robert C , Munoz A , Moreau D , et al. Clinical implementation of deep‐learning based auto‐contouring tools–Experience of three French radiotherapy centers. Cancer/Radiothérapie. 2021;25(6‐7):607‐616. doi:10.1016/J.CANRAD.2021.06.023 34389243 10.1016/j.canrad.2021.06.023

[30] Vandewinckele L , Claessens M , Dinkla A , et al. Overview of artificial intelligence‐based applications in radiotherapy: recommendations for implementation and quality assurance. Radiother Oncol. 2020;153:55‐66. doi:10.1016/J.RADONC.2020.09.008 32920005 10.1016/j.radonc.2020.09.008

[31] Dzindolet MT , Peterson SA , Pomranky RA , Pierce LG , Beck HP . The role of trust in automation reliance. Int J Hum Comput Stud. 2003;58(6):697‐718. doi:10.1016/S1071‐5819(03)00038‐7

[32] Skitka LJ , Mosier K , Burdick MD . Accountability and automation bias. Int J Hum Comput Stud. 2000;52(4):701‐717. doi:10.1006/IJHC.1999.0349

[33] Bobek S , Nalepa GJ . Introducing uncertainty into explainable AI methods. Lect Notes Comput Sci (including Subser Lect Notes Artif Intell Lect Notes Bioinformatics). 2021;12747:444‐457. doi:10.1007/978‐3‐030‐77980‐1_34. LNCS.

[34] Masalonis AJ . Effects of training operators on situation‐specific automation reliability. Proc IEEE Int Conf Syst Man Cybern. 2003;2:1595‐1599. doi:10.1109/ICSMC.2003.1244640

[35] Wang T , Tam J , Chum T , et al. Evaluation of AI‐based auto‐contouring tools in radiotherapy: a single‐institution study. J Appl Clin Med Phys. 2025;26(4):e14620. doi:10.1002/ACM2.14620 39837647 10.1002/acm2.14620 PMC11969097

[36] Duan J , Bernard M , Downes L , et al. Evaluating the clinical acceptability of deep learning contours of prostate and organs‐at‐risk in an automated prostate treatment planning process. Med Phys. 2022;49(4):2570‐2581. doi:10.1002/MP.15525 35147216 10.1002/mp.15525

[37] Starke A , Poxon J , Patel K , et al. Clinical evaluation of the efficacy of limbus artificial intelligence software to augment contouring for prostate and nodes radiotherapy. Br J Radiol. 2024;97(1158):1125‐1131. doi:10.1093/bjr/tqae077 38627245 10.1093/bjr/tqae077 PMC11135797

[38] Shen J , Tao Y , Guan H , et al. Clinical validation and treatment plan evaluation based on autodelineation of the clinical target volume for prostate cancer radiotherapy. Technol Cancer Res Treat. 2023;22(1):1‐8. doi:10.1177/15330338231164883 10.1177/15330338231164883 PMC10064523 36991566

[39] Guo H , Wang J , Xia X , et al. The dosimetric impact of deep learning‐based auto‐segmentation of organs at risk on nasopharyngeal and rectal cancer. Radiat Oncol. 2021;16(1):113. doi:10.1186/s13014‐021‐01837‐y 34162410 10.1186/s13014-021-01837-y PMC8220801

[40] Dinç SÇ , Üçgül AN , Bora H , Şentürk E . The dosimetric impacts of ct‐based deep learning autocontouring algorithm for prostate cancer radiotherapy planning dosimetric accuracy of DirectORGANS. BMC Urol. 2025;25(1):190. doi:10.1186/S12894‐025‐01875‐8 40753235 10.1186/s12894-025-01875-8 PMC12317517

[41] Arjmandi N , Mosleh‐Shirazi MA , Mohebbi S , et al. Evaluating the dosimetric impact of deep‐learning‐based auto‐segmentation in prostate cancer radiotherapy: insights into real‐world clinical implementation and inter‐observer variability. J Appl Clin Med Phys. 2025;26(3):e14569. doi:10.1002/ACM2.14569 39616629 10.1002/acm2.14569 PMC11905246

